# Growth Performance and Recovery of Nosocomial *Aspergillus* spp. in Blood Culture Bottles

**DOI:** 10.3390/microorganisms10102026

**Published:** 2022-10-13

**Authors:** Salvatore Pasqua, Francesco Monaco, Francesca Cardinale, Simone Bonelli, Pier Giulio Conaldi, Danilo D’Apolito

**Affiliations:** 1 Unità Prodotti Cellulari (GMP), Fondazione Ri.MED c/o IRCCS-ISMETT, Via E. Tricomi 5, 90127 Palermo, Italy; 2 Unità di Medicina di Laboratorio e Biotecnologie Avanzate, IRCCS-ISMETT (Istituto Mediterraneo per i Trapianti e Terapie ad Alta Specializzazione), Via E. Tricomi 5, 90127 Palermo, Italy; 3 Department of Biological, Chemical and Pharmaceutical Sciences and Technologies (STEBICEF), University of Palermo, Viale delle Scienze, Edificio 16, 90128 Palermo, Italy; 4 Proteomics Group of Fondazione Ri.MED, Department of Research IRCCS ISMETT, Via Ernesto Tricomi 5, 90145 Palermo, Italy

**Keywords:** Aspergillus, recovery, blood culture bottles, BACTEC, BacT/Alert, automated culture system, sterility, analytical method, infections, European pharmacopeia

## Abstract

Theoretically, *Aspergillus* spp. grow in culture media, but frequently, blood cultures of patients with invasive Aspergillosis are negative, even if until now, the reasons are not clear. This aspect underlines the lack of a good strategy for the cultivation and isolation of *Aspergillus* spp. In order to develop a complete analytical method to detect *Aspergillus* in clinical and pharmaceutical samples, we investigated the growth performance of two blood culture systems versus the pharmacopeia standard method. At <72 h, all test systems showed comparable sensitivity, about 1–2 conidia. However, the subculture analysis showed a suboptimal recovery for the methods, despite the positive growth and the visualization of the “*Aspergillus* balls” in the culture media. To investigate this issue, we studied three different subculture approaches: (i) the use of a sterile subculture unit, (ii) the use of a sterile subculture unit and the collection of a larger aliquot (100 µL), following vigorous agitation of the vials, and (iii) to decapsulate the bottle, withdrawing and centrifuging the sample, and aliquot the pellet onto SDA plates. Our results showed that only the third procedure recovered *Aspergillus* from all positive culture bottles. This work confirmed that our strategy is a valid and faster method to culture and isolate *Aspergillus* spp. from blood culture bottles.

## 1. Introduction

*Aspergillus* spp. are ubiquitous fungi with a worldwide distribution in nature and are frequently isolated from air, devices and surfaces in hospital areas. *Aspergillus* species may lead to a variety of allergic reactions and life-threatening systemic infections in humans. Aspergillus infections (AI) are associated with high mortality rates in transplanted and immunocompromised patients [1,2,3], and recent studies indicate that about 50% of AI cases are detected only in postmortem [4,5].

The diagnosis of AI involves radiological, microbiological, molecular and serological tests [1,2,3], but only the isolation of Aspergillus colonies in a microbiological culture facilitates antifungal susceptibility testing. Nevertheless, fungal infection detection has always been a difficult challenge for the diagnostic laboratory [6,7,8,9] since blood culture analysis often returns negative results or, in the case of positivity, it is difficult to obtain the fungal recovery to perform an antifungal susceptibility test [10,11,12,13].

Similar to clinical blood cultures, the problem of *Aspergillus* spp. isolation also involves Advanced Therapy Medicinal Products (ATMP) quality control [14], which we perform in our laboratory. ATMPs are increasingly important forms of treatment for different kinds of diseases, including various types of cancer and immunological diseases. It is mandatory to detect Aspergillus contamination of these products prior to patient infusion, to avoid the onset of AI.

To detect fungal contaminations of these products in our laboratory, we perform microbiological culture methods, as recommended by the United States Pharmacopeia (USP) (USP <71> Sterility Tests) and European Pharmacopeia (Ph. Eur.) (Ph. Eur. 2.6.1 Sterility and Ph. Eur. 2.6.27 Microbiological Control of Cellular Product) being the gold standard.

In recent years, automated blood culture systems such as BACTEC or BacT/Alert have been used in place of the pharmacopeial compendial method to test ATMPs [15,16,17,18,19,20].

Despite their ability to detect microorganisms defined in international regulations, further and continuous studies are necessary to understand their ability to detect the presence of most nosocomial and cGMP environmental microorganisms or common contaminants of biological fluids and biopharmaceutical products [6,7,8,9].

In this regard, we observed that in preliminary studies with nosocomial fungal isolates, several authors reported a deficiency in the performance of such systems and described that *Aspergillus* spp. recovery from blood cultures is difficult, and most of the time it is considered a contaminant and interpreted as a false positive result [10,11,12,13]. In particular, C. Rosa et al. [13] showed that the routine subculture strategy employed by clinical laboratories rarely recovers *Aspergillus* spp. from blood cultures and demonstrated a new subculture approach to increase *Aspergillus* recovery.

In light of these indications, this study aimed to investigate *Aspergillus* spp. detection by automated blood culture methods, comparing the results with pharmacopeial compendial method results and improving the process of transferring subculture onto solid media to increase our diagnostic ability for these nosocomial species.

## 2. Materials and Methods

### 2.1. Nosocomial Isolates and Reference Control Strain

Four clinical isolates were tested. Each isolate was obtained by environmental monitoring of hospital surfaces and identified by MALDI-TOF.

The four microorganisms included: *Aspergillus fumigatus*, *Aspergillus flavus*, *Aspergillus niger* and *Aspergillus terreus*. Moreover, *Aspergillus brasiliensis* ATCC 16,404 (Microbiologics St. Cloud, MN, USA) was used as a reference control (Table 1).

### 2.2. Inoculum Adjustment

The size of the inoculum from which to prepare the dilutions was adjusted between 1.0 × 10^6^ and 5.0 × 10^6^ spores/mL by microscopic enumeration with a cell-counting hematocytometer. Stock suspensions of all microorganisms and subsequent dilutions were prepared in peptone water (Becton Dickinson [BD], Franklin Lakes, NJ, USA). All suspensions were quantified by plating on Sabouraud dextrose agar (SDA) plates (BD). The inocula were vortexed for 15 s and then dilutions were performed to obtain five microbial suspensions containing: 25–50, 5–10, 2–5, 1–2, and 0–1 CFU per 100 μL.

The titer of the microbial suspensions was verified by plating in triplicate 100 μL on SDA plates. The plates were incubated at 30–35 °C and the number of colonies was determined as soon as possible after the observation of visible growth and within 5 days. ATCC reference strain was cultured as indicated by the manufacturer.

### 2.3. Experimental Strategy

To compare the test methods, we performed the experiments in parallel. For the sample preparation, sterile 25 mL conical bottom tubes (Eppendorf, Hamburg, Germany) and 21G needles (BD) were used.

For each microorganism, the results obtained with the BacT/Alert method (Biomeriéux, Marcy-l’Étoile, France), the BACTEC method (BD) and the gold standard method were compared to evaluate their effectiveness.

For each microorganism and each method under analysis, we performed four independent experiments creating a total of 60 bottles inoculated with five microbial suspensions containing 25–50, 5–10, 2–5, 1–2, and 0–1 CFU.

For each experiment, the determination of viability, purity and titer was evaluated by plating 100 µL of each suspension in triplicate on Trypticase soy agar (TSA) and Sabouraud dextrose agar (both from BD). TSA and SDA plates were incubated at 30 to 35 °C and 20 to 25 °C, respectively. CFUs were quantified after 3–5 days. The titer of each suspension was confirmed by calculating the arithmetic mean of CFU values obtained on the SDA plates.

Three recovery approaches were used for the subculture in a solid medium: (i) the routine method, using a sterile airway needle/subculture unit (Biomeriéux) and collecting 2 drops onto SDA plates, (ii) using a sterile airway needle/subculture unit and collecting a larger aliquot (100 µL), following vigorous agitation of the vials, and (iii) decapsulating the bottle, withdrawing and centrifuging the sample, and collecting 100 µL of the pellet onto SDA plates.

### 2.4. Sterility Compendial Method

The compendial Ph. Eur. method was performed as described elsewhere [21] and in accordance with international pharmacopeias.

Briefly, we examined the growth properties of the five strains (Table 1) in TSB (BD, catalog No. 299416, 100 mL bottle, septum/screw cap) incubated at 22.5 °C.

Growth was assessed daily by observing turbidity for 14 days.

### 2.5. Automated Blood Culture Methods

Among the different media available, we chose to use iAST bottles (Biomeriéux) for BacT/Alert 3D 60 and Aerobic/F (BD) and Mycosis-IC/F (BD) bottles for the BACTEC system.

The growth of microorganisms was performed at 35–37 °C, as recommended by the specific monographs (Ph. Eur. 2.6.27 Microbiological Control of Cellular Product and Ph. Eur. 5.1.6 Alternative methods for microbiological control quality).

BacT/Alert and BACTEC bottles were automatically monitored by the software instrument, which reported the time to detection (TTD), i.e., the time at which the instrument detected microorganism growth for the first time. A visual inspection of the BacT/Alert and BACTEC bottles was performed at the growth detection and the end of the 14-day incubation period for the negative bottles.

### 2.6. Subculture

Each bottle in which growth was evident was subcultured with the three approaches detailed above, in Trypticase soy agar (TSA) and Sabouraud Dextrose Agar (SDA) and incubated at 30 to 35 °C and 20 to 25 °C, respectively, to evaluate the identity and purity of the corresponding organism. All microorganisms and all positive bottles were confirmed by Gram stain morphology and MALDI-TOF analysis.

After 14 days, the negative samples were subcultured in the same manner to ensure that they were not false negatives.

### 2.7. Parameters under Investigation

In this study, we investigated the following parameters: specificity, detection limit, ruggedness, repeatability, and cross-reaction contamination [22].

### 2.8. Specificity

To evaluate the ability of the method to detect the specific presence of the microorganism present in the test, each operator tested the microbial culture media inoculating one microorganism at a time, with a concentration of 25–50 CFU, and tested the media for growth ability.

### 2.9. Detection Limit

To investigate the lowest number of fungi that each analytical method could detect, we performed four independent analyses, testing three repetitions of microbial suspensions of 25–50, 5–10, 2–5, 1–2, and 0–1 CFU. We chose a detection limit of 50% of total inoculated bottles [21,23].

### 2.10. Ruggedness and Repeatability

The ruggedness of an analytical method can generally be described as the ability to reproduce an analytical method in different laboratories or different circumstances without the occurrence of unexpected differences in the obtained results. To evaluate this parameter for each method, the experiments described above were conducted on four different days by four different operators, each using a different batch of microbiological culture medium.

Repeatability is a measure of the ability of the method to generate similar results for multiple preparations of the same sample made under the same experimental conditions. To demonstrate this parameter, each operator performed three different inoculations of each microbial suspension dilution on the same day.

### 2.11. Technical Cross-Contamination

Technical cross-contamination is the unwanted transfer of other microorganisms into the sample. To ensure that our experimental procedures were not prone to cross-reaction contaminations, for each operator and each microorganism, the evaluation involved the simultaneous handling and processing of twelve positive and five negative samples for the bottles and six positive and two negative samples for the plates.

### 2.12. Comparative Data Analysis

A comparison of the three systems regarding the growth of microorganisms at low-level inoculation (25–50 CFU) was determined by the χ2 test. We compared the number of positive cultures detected with each method. The normality of data and homogeneity of variances was tested with a graphical approach, using a Q-Q plot and *F*-test statistics, respectively. The *t*-test was used to compare the TTD values needed to detect microbial growth with the methods. STATA 15.1 statistical software (StataCorp, College Station, TX, USA) was used for all statistical analysis. We considered a value of *p* < 0.05 statistically significant.

## 3. Results

### 3.1. Specificity

Specificity for each method was evaluated by performing a growth promotion test (GPT) inoculating 25–50 colony-forming units (CFU) of fungi, as detailed in Table 1.

The results are summarized in Table 2. For each medium, we determined its ability to promote the growth of each microorganism.

To demonstrate the specificity, we analyzed the results obtained at the end of a bottle’s incubation. We did not find any difference between the three analytical methods in terms of the promotion of the growth of the five *Aspergillus* used. In each experiment and each media, the complete recovery of *Aspergillus* was observed.

### 3.2. Detection Limit

Table 3 summarizes the results analyzed to determine the detection limit.

Using TSB, iAST, Aerobic F, and Mycosis media, we observed detection limits of 1–2 CFU for the five *Aspergillus* tested.

In each experiment, for each microbial suspension, the actual number of CFU was confirmed on appropriate solid media plates and agreed with the range of CFU used in this validation.

### 3.3. Ruggedness and Repeatability

To evaluate the ruggedness and the repeatability, we analyzed the results obtained from GPTs, where the bottle’s media were inoculated with 25–50 CFU of the tested *Aspergillus* species. Table 2 summarizes the results obtained from four independent operators working on four different days and using different media lots. For each method and each *Aspergillus* spp., all inoculated bottles showed growth, demonstrating that all three methods produced robust and reproducible results.

### 3.4. Technical Cross-Contaminations

No cross-contamination of the negative samples occurred in any of the experiments performed during the method validation.

### 3.5. Comparative Data Analisys

To perform the comparative data analysis, we considered the values of the inoculations using 25–50 CFU, where the growth of all cultured microorganisms displayed less variability.

#### 3.5.1. Comparative Analysis of the Growth Promotion Test (GPT) for the Three Systems

The results in Table 2 show that the three methods, with the media used, were equivalent for the growth promotion of all five *Aspergillus*.

#### 3.5.2. Time to Detection (TTD)

**Figure 1:**
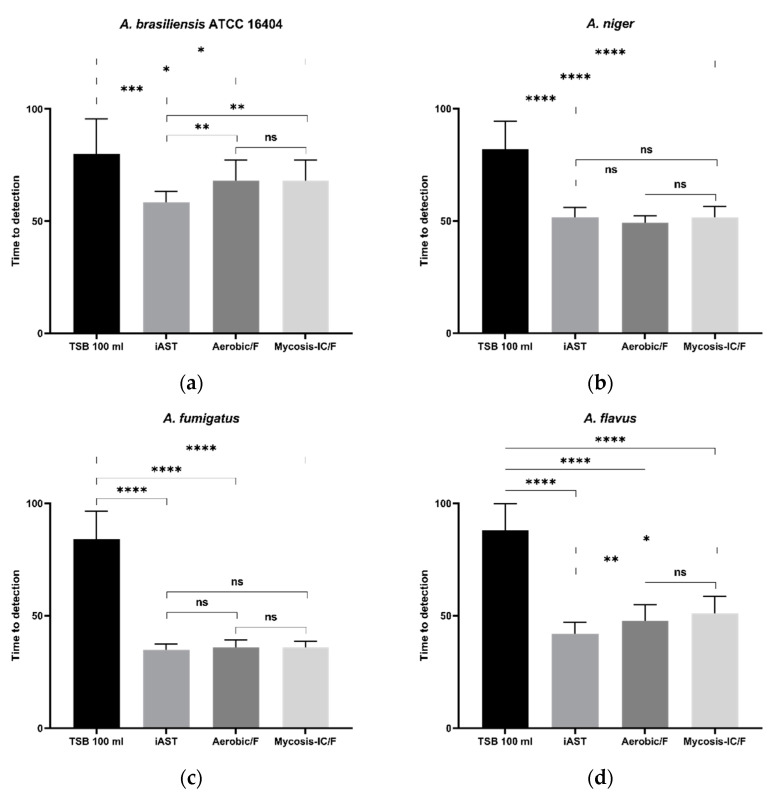

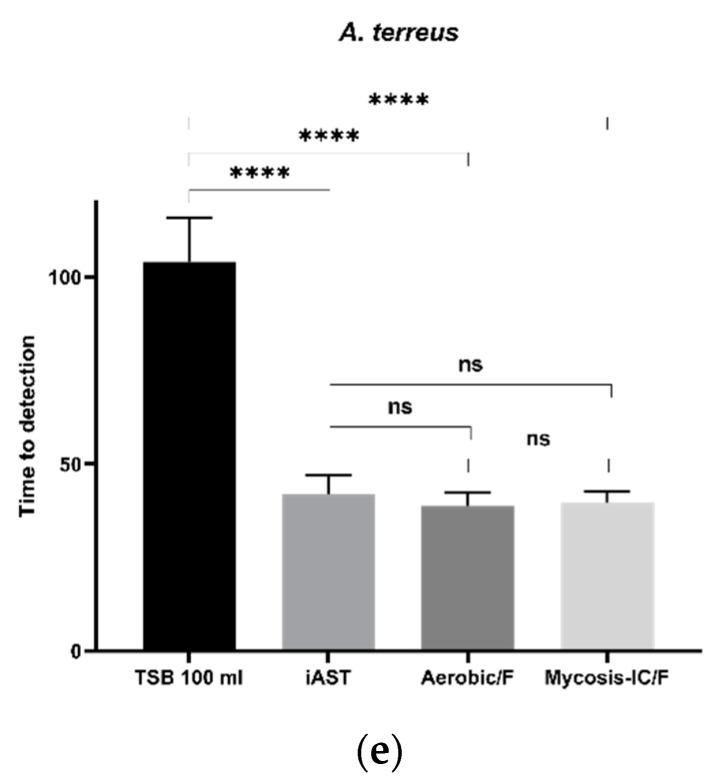
Time to Detection (TTD) of *Aspergillus* spp. with the manual method (TSB), the BacT/Alert 3D 60 system (iAST), and the BACTEC 9020 system (Aerobic/F or Mycosis-IC/F). (**a**) *A. brasiliensis*; (**b**) *A. niger*; (**c**) *A. fumigatus*; (**d**) *A. flavus*; (**e**) *A. terreus*; In each figure, the bar represents the standard deviation of the mean. Symbols above comparative bars denoting statistically significant differences among groups as follows: ns: no significant difference (*p* > 0.05), * = *p* < 0.05, ** = *p* < 0.01, *** = *p* < 0.01, **** = *p* < 0.001.

As detailed in Table 2 and Figure 1, all *Aspergillus* spp. grew faster in the alternative bottle media than in TSB.

However, some *Aspergillus* showed a significant lower TTD when diverse blood culture media were used, such as *A. brasiliensis* ATCC 16,404 (Figure 1a) showed a lower TTD in iAST than in Aerobic/F (*p* = 0.0045) or than in Mycosis-IC/F (*p* = 0.0045). Similar results were observed for *A. flavus* (Figure 1d) which grew faster in iAST than in Aerobic/F (*p* = 0.0022) or Mycosis-IC/F (*p* = 0.0375).

No statistically significant differences were observed between iAST and Aerobic/F and Mycosis-IC/F regarding the detection of *A. niger* (Figure 1b), *A. fumigatus* (Figure 1c), and *A. terreus* (Figure 1e).

### 3.6. Recovery of Aspergillus *spp.* on Solid Culture Medium

Three distinct procedures were used for the recovery of *Aspergillus* spp. from the culture bottles: (i) the first used a sterile airway needle/subculture unit for the collection of two drops of culture medium onto solid culture media, (ii) the second used the same subculture unit for the collection of 100 µL, after vigorous agitation of the culture vials (approx. 15 s) to fragment fungal balls or disperse the spore and (iii) the third involved decapsulating the bottles, collecting and centrifuging the sample and plating 100 µL of the pellet on SDA and TSA plates. The plates were examined for up to 120 h.

Results of 25–50 CFU inoculations and recovery on solid culture medium are summarized in Table 4.

The first and the second approaches resulted in the occasional recovery of *Aspergillus* spp., with a recovery of less than 35%, even when these bottles had visible “fungal balls”. Similar results were obtained from positive vials initially inoculated with 5–10 CFU per inoculum. Moreover, *Aspergillus* spp. were rarely cultured from positive vials with 2–5 CFU per inoculum or lower concentrations (Data not shown).

The third procedure succeeded in the isolation of all five *Aspergillus* spp. from all prepositive culture vials detected by the three methods, even with the lowest concentration of CFU.

## 4. Discussion

*Aspergillus* spp. are ubiquitous fungi with a worldwide distribution in nature and are frequently isolated from air, devices and surfaces in hospital areas.

In recent years, the incidence and epidemiology of invasive *Aspergillus* infections have augmented, due to the continued increase in patients with predisposing risk factors (e.g., transplantation, chemotherapy, HIV infection, and immunosuppression) [1,24,25,26,27], and is often associated with high rates of mortality and morbidity [1,2,3].

Hospital cleaning is an important intervention in the control of AI, but mold removal in hospitals and GMP environments by disinfectants is difficult and requires strong validated procedures and the continuous training of personnel [28,29,30,31,32,33].

Different species of *Aspergillus* can cause disease; the most common species of *Aspergillus* causing human disease are *Aspergillus fumigatus*, *Aspergillus flavus*, *Aspergillus niger,* and *Aspergillus terreus* [24,34].

Diagnosing AI is an ongoing and difficult challenge and requires a high level of collaboration between clinicians and diagnostic laboratory staff.

Diagnostic laboratories use radiological, microbiological, molecular and serological tests to detect *Aspergillus* spp. in biological samples, but only the isolation of colonies in microbiological culture facilitates the determination of antifungal susceptibility patterns [3,10].

In the last 20 years, the use of automated culture systems for mold detection has undergone a significant increase and several studies have been carried out to improve the detection of these microorganisms from blood samples and biological matrices.

C. Rosa et al. [13], showed that the traditional method for the subculture of presumptively positive culture bottles did not result in the recovery of *Aspergillus* spp., even when such vials presented visible “fungal balls”. Similar results were obtained in our laboratory in preliminary tests with blood culture media (data not shown).

Moreover, different authors reported that *Aspergillus* spp. recovered from blood cultures are often considered a contaminant and interpreted as false positive results [10,11,12,13] and this could be explained by the lack of recovery of Aspergillus conidia from the majority of blood culture bottles, as mentioned by C. Rosa et al. [13], leading to an underestimation of AI.

The present study was planned considering the serious dangers posed by Aspergillus in the infection of transplanted and/or immunocompromised patients. It is essential to determine an effective recovery protocol capable of isolating these microorganisms on solid media, from which to perform antifungal susceptibility tests. This will be essential to plan adequate diagnostic procedures that result in proper antifungal prophylaxis.

Initially, to investigate this topic and in order to define an effective quality control analytical method capable of recovering *Aspergillus* contaminations in our samples, we chose to study the growth performance of two automatic systems for blood cultures versus the Ph. Eur. gold standard method. As suggested by the ICH Q2 guideline, for these qualitative analytical methods, we investigated the following parameters: specificity, detection limit, ruggedness, repeatability, and cross-reaction contamination [22].

Specificity was confirmed by the ability of all selected media to promote the growth of all *Aspergillus* spp. No variable results were observed even when the three methods were performed by four independent operators, in four days, and using different media lots, confirming the ruggedness and repeatability of the gold standard and the two automated culture methods (Table 3). Simultaneous handling and processing of contaminated and negative samples showed no cross-reaction contamination since no false negatives or false positives were found, as expected. Regarding detection limit, the three methods showed, for all five *Aspergillus* spp., a sensitivity of 1–2 conidia for 8 mL of inoculum (Table 3).

Overall, the two automated culture methods satisfied all the requirements stated by the Ph. Eur. for an alternative method regarding specificity, ruggedness, detection limit, repeatability, and cross-reaction contamination.

The analysis of the TTDs of each *Aspergillus* showed that each microorganism grew faster in the alternative bottle media than in TSB (Table 2 and Figure 1). Furthermore, the tested *Aspergillus* grew similarly in the microbiological media of the two automated systems except for *A. brasiliensis* and *A. flavus* which grew faster in the BacT/Alert medium than both BACTEC media.

As expected, different *Aspergillus* have different TTDs, confirming the need to test other species that may contaminate hospital surfaces, clinical samples, or pharmaceutical products, as suggested in Ph. Eur. 2.6.27.

These results demonstrated that automated culture systems may allow sensitive and fast detection of the most common species of *Aspergillus* causing human disease, representing also the flora from the hospital environment, with a better performance of BacT/Alert versus the BACTEC system.

Further studies are still necessary to confirm the growth performance of such automated methods for the detection of *Aspergillus* in blood, pharmaceutical and other samples.

Subsequently, our protocol investigated the recovery method of *Aspergillus* spp. from the bottles’ media, since the traditional method for the subculture of presumptively positive culture bottles did not result in an optimal recovery performance [13].

To investigate how to overcome this issue, we compared three recovery approaches: (i) the routine method, using a sterile airway needle/subculture unit and collecting two drops onto SDA plates, (ii) using a sterile airway needle/subculture unit and collecting a larger aliquot (100 µL), following vigorous agitation of the vials, and (iii) decapsulating the bottle, withdrawing and centrifuging the sample, and collecting 100 µL of the pellet onto SDA plates.

In the first approach, we observed that the mycelium did not pass through the 20-gauge needle, and although in the second we modified the procedure by shaking the bottles vigorously, again, the mycelium did not pass and/or agglutinate like cotton candy, forming a blockage in the needle most of the time. In the third procedure, the direct withdrawal of the medium after removing the cap solved this problem.

In conclusion, our results (Table 4) showed that the first two procedures were suboptimal in recuperating the microorganisms on solid media and only the third procedure was able to recover all five *Aspergillus* spp. from all positive culture bottles, even with the lowest concentrations of CFU.

To the best of our knowledge, this is the first study investigating these critical issues using these four nosocomial aspergilli simultaneously. In light of the results obtained, for patients with suspected AI or ATMPs samples, we propose a diagnostic procedure using three bottles (aerobe and anaerobe plus a third bottle only for mold detection using our described recovery approach) rather than two (aerobe and anaerobe), which could be more efficient, easy and reliable than current the strategy used in clinical laboratories (Table S1).

## 5. Conclusions

In conclusion, our analytical strategy is an example of an easy, efficient and reliable method to detect and isolate *Aspergillus* spp. from clinical and ATMP samples without the need for preanalytical processing.

We believe that our work is of interest to clinicians and researchers involved in *Aspergillus* spp. detection using automated blood culture systems, because this approach improves the chances of recovering *Aspergillus* from liquid culture bottles to perform identification and antimicrobial susceptibility testing which is fundamental for the targeted therapy setting.

Moreover, we hope that the application of this analytical strategy and the new subculture approach could improve the evaluation of *Aspergillus* fungemia in AI in critical patients and ameliorate *Aspergillus* spp. detection in Advanced Medicinal Therapy Products.

## Figures and Tables

**Table 1:** The Aspergillus strains used in this work.

Clinical Isolates	Reference Strain
*Aspergillus fumigatus*	*Aspergillus brasiliensis* ATCC 16404
*Aspergillus flavus* *Aspergillus niger* *Aspergillus terreus*	

**Table 2:** Growth Promotion Test. Specificity analysis and TTD.

Aspergillus	Bottle Media	Recovery	Recovery TTD (h) ± SD
*A. brasiliensis* (ATCC 16404)	TSB	12/12	80 ± 15.6
	iAST	12/12	58.4 ± 4,8
	Aerobic/F	12/12	68 ± 9.3
	Mycosis-IC/F	12/12	68 ± 9.3
*A. niger*	TSB	12/12	79.5 ± 11.3
	iAST	12/12	51.6 ± 4.4
	Aerobic/F	12/12	49.2 ± 3.1
	Mycosis-IC/F	12/12	51.6 ± 4.7
*A. fumigatus*	TSB	12/12	84 ± 12.5
	iAST	12/12	34.8 ± 2.6
	Aerobic/F	12/12	35.8 ± 3.5
	Mycosis-IC/F	12/12	35.8 ± 2.8
*A. flavus*	TSB	12/12	87.2 ± 12.1
	iAST	12/12	41.54 ± 4.8
	Aerobic/F	12/12	48.9 ± 6
	Mycosis-IC/F	12/12	52.6 ± 5.4
*A. terrus*	TSB	12/12	104 ± 11.8
	iAST	12/12	42 ± 4.9
	Aerobic/F	12/12	38.3 ± 3.7
	Mycosis-IC/F	12/12	39.6 ± 3.2

TTD, Time To Detection; SD, Standard Deviation.

**Table 3:** Positive detection of *Aspergillus* spp. by specific bottle media.

Range of Total CFU Inoculated		25–50	5–10	2–5	1–2	0–1
Aspergillus	Bottle Media	Recovery	Recovery	Recovery	Recovery	Recovery
*A. brasiliensis* (ATCC 16404)	TSB	12/12	12/12	11/12	9/12	2/12
	iAST	12/12	12/12	12/12	9/12	0/12
	Aerobic/F	12/12	12/12	10/12	10/12	1/12
	Mycosis-IC/F	12/12	12/12	12/12	9/12	1/12
*A. niger*	TSB	12/12	12/12	12/12	10/12	1/12
	iAST	12/12	12/12	12/12	9/12	0/12
	Aerobic/F	12/12	12/12	11/12	10/12	3/12
	Mycosis-IC/F	12/12	12/12	10/12	8/12	0/12
*A. fumigatus*	TSB	12/12	12/12	12/12	9/12	0/12
	iAST	12/12	12/12	11/12	10/12	2/12
	Aerobic/F	12/12	12/12	11/12	9/12	0/12
	Mycosis-IC/F	12/12	12/12	11/12	10/12	1/12
*A. flavus*	TSB	12/12	12/12	11/12	9/12	2/12
	iAST	12/12	12/12	10/12	9/12	0/12
	Aerobic/F	12/12	12/12	12/12	9/12	1/12
	Mycosis-IC/F	12/12	12/12	10/12	10/12	1/12
*A. terrus*	TSB	12/12	12/12	10/12	9/12	2/12
	iAST	12/12	12/12	10/12	10/12	0/12
	Aerobic/F	12/12	12/12	11/12	9/12	1/12
	Mycosis-IC/F	12/12	12/12	10/12	10/12	1/12

**Table 4:** Recovery of *Aspergillus* spp. from solid medium.

Range of Total CFU Inoculated		25–50			
Aspergillus	Bottle Media	Positive Detection in Liquid Medium	Recovery from Solid Medium		
			First Procedure ^1^	Second Procedure ^2^	Third Procedure ^3^
*A. brasiliensis* (ATCC 16404)	TSB	12/12	1/12	2/12	12/12
	iAST	12/12	0/12	2/12	12/12
	Aerobic/F	12/12	1/12	4/12	12/12
	Mycosis-IC/F	12/12	0/12	3/12	12/12
*A. niger*	TSB	12/12	1/12	3/12	12/12
	iAST	12/12	0/12	3/12	12/12
	Aerobic/F	12/12	2/12	2/12	12/12
	Mycosis-IC/F	12/12	0/12	3/12	12/12
*A. fumigatus*	TSB	12/12	1/12	3/12	12/12
	iAST	12/12	1/12	2/12	12/12
	Aerobic/F	12/12	0/12	4/12	12/12
	Mycosis-IC/F	12/12	1/12	3/12	12/12
*A. flavus*	TSB	12/12	2/12	3/12	12/12
	iAST	12/12	0/12	2/12	12/12
	Aerobic/F	12/12	0/12	2/12	12/12
	Mycosis-IC/F	12/12	1/12	3/12	12/12
*A. terrus*	TSB	12/12	1/12	3/12	12/12
	iAST	12/12	1/12	2/12	12/12
	Aerobic/F	12/12	0/12	2/12	12/12
	Mycosis-IC/F	12/12	0/12	2/12	12/12

^1^ The first procedure used a sterile airway needle/subculture unit for the collection of two drops of culture medium onto solid culture media; ^2^ The second procedure used the same sterile airway needle/subculture unit for the collection of 100 µL onto solid culture media, after vigorous agitation of the culture vials (approx. 15 s); ^3^ The third procedure involved decapsulating the bottles, collecting and centrifuging the sample, and 100 µL of the pellet was plated onto solid media.

## Data Availability

Not applicable.

## Supplementary Materials

The following supporting information can be downloaded at: https://www.mdpi.com/article/10.3390/microorganisms10102026/s1, Table S1: Comparison of “In house analytical strategy” with the current methods.
