# Supplementary material for: Growth Performance and Recovery of Nosocomial Aspergillus spp. in Blood Culture Bottles
Source: Microorganisms. 2022 Oct 13;10(10):2026. doi: 10.3390/microorganisms10102026 (PMC9608713; doi:10.3390/microorganisms10102026)
Supplement: Supplementary file 1 [file microorganisms-10-02026-s001.zip › Table S1.pdf]

Table S1. Comparison of "In house analytical strategy" with the current methods.

Aspect of comparison	Strategy		Improvement
	**Current method (2 Bottles)**	**In house 3 Bottles strategy**	
Growth of Aspergillus in bottles	Yes	Yes	Equal
% Recovery of Aspergillus onto Solid Media	< 35%	100%	Efficiency and Reliability
Necessity to repeat the sample collection to evaluate fungal infection and use of a different approach as isolator tube	Yes	No	Ease, Efficiency Reliability and Cheapness
Availability of a dedicated bottle for the complete recovery of Aspergillus separate from the "Blood culture set" used for the detection of other microorganisms.	No	Yes	Efficiency
Days of complete procedure	>4-5	<4	Efficiency and Reliability
